# Utero-vaginal anastomosis in cases of cervical malformations: long-term follow-up and fertility challenges

**DOI:** 10.1007/s00404-022-06858-w

**Published:** 2022-11-28

**Authors:** Reham Fouad, Mohamed Zayed

**Affiliations:** Obstetrics and Gynecology Department, Faculty of Medicine, Cairo University, 1 Al Sarayh St., Al Manyal, Cairo, Egypt

**Keywords:** Utero-vaginal anastomosis, Cervical malformations, Cervical dysgenesis, Cervical agenesis

## Abstract

**Purpose:**

To study the long-term results of utero-vaginal anastomosis in cases of cervical malformations.

**Methods:**

This is a retrospective cohort study. Nine patients presented with cryptomenorrhea due to cervical malformations (5 patients with cervical agenesis and vaginal aplasia, 2 patients with cervical agenesis and upper vaginal aplasia, and two patients with cervical dysgenesis in form of cervical obstruction). Five patients had utero-vaginal anastomosis (UVA) with McIndoe vaginoplasty. Four patients had UVA without vaginoplasty. Follow-up was done by transabdominal and/or transvaginal ultrasound monthly for the first 3 months then every 6 months thereafter for a duration that ranged from 15 to 82 months. The main outcome measures are achieving menstruation, dysmenorrhea, pelvic inflammatory disease (PID), needed interventions after primary surgery, infertility, and pregnancy rate.

**Results:**

Nine (100%) patients achieved menstruation, one (12%) experienced severe dysmenorrhea, two (22%) had PID, seven (78%) needed dilatation of the anastomosis site, three (33%) needed reoperation, nine (100%) had primary infertility, two (28.5%) achieved clinical pregnancy, and only one (14%) ended by live birth.

**Conclusion:**

Conservative surgery for cervical malformation is a promising choice for relieving the obstructive symptoms. Regular dilatation is recommended. Pregnancy is a remote hope that is hindered by many challenges.

## What does this study add to the clinical work

**Table:** 

Conservative surgery for management of cervical malformation is a promising choice for relieving the obstructive symptoms but pregnancy is a remote hope. Regular dilatation of the anastomosis site is recommended.	

## Introduction

The prevalence of congenital cervical agenesis or dysgenesis ranges from 1/80,000 to 1/100,000, and in about 50% of cases, it is associated with congenital vaginal aplasia [1]. For many decades, the classic management for congenital cervical malformations was hysterectomy [2]. However, with the growing experience in reconstructive surgeries and in assisted reproductive technology (ART), the hopes of fertility for such challenging cases have been increasing; thus, conservative surgeries for management of cervical malformations have been recently encouraged [3], 4. Information about long-term follow-up and reproductive performance of these cases is lacking. In this study, we aim to analyze and discuss long-term outcome, complications and fertility challenges following conservative surgical management of cervical malformations.

## Materials and methods

**Fig. 1:**
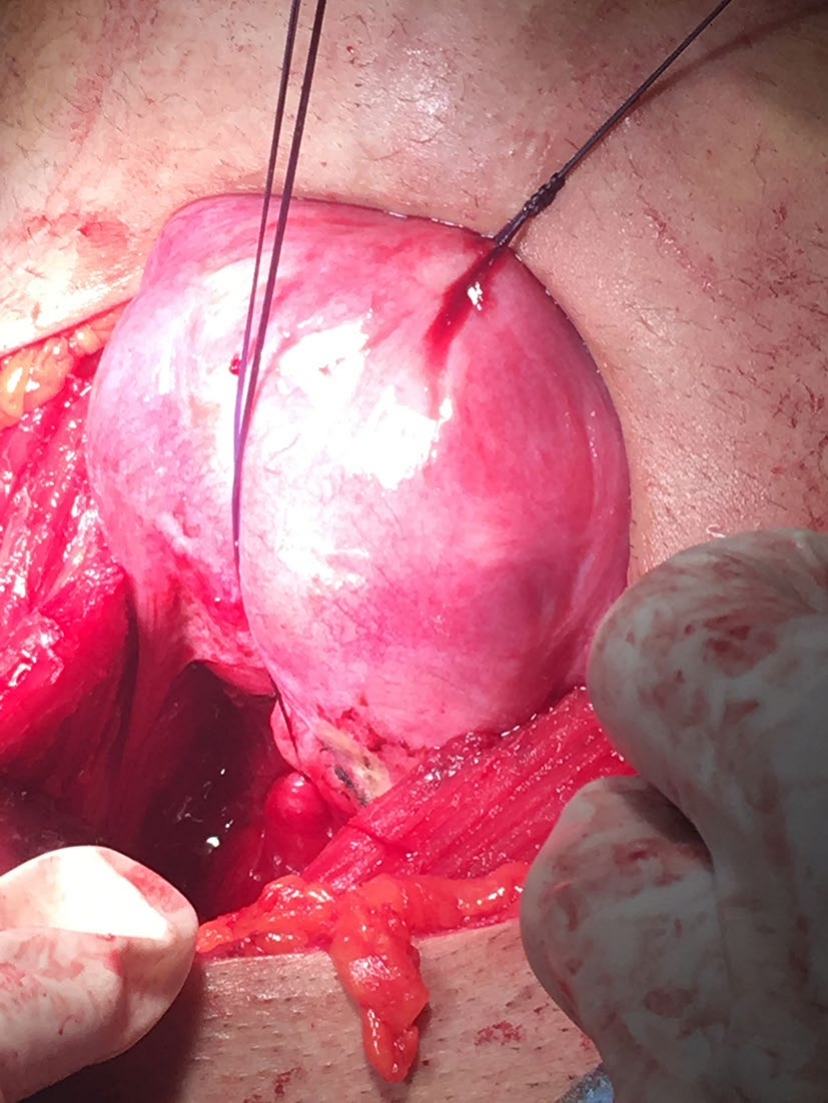
The cord-like cervical tissue is identified

**Fig. 2:**
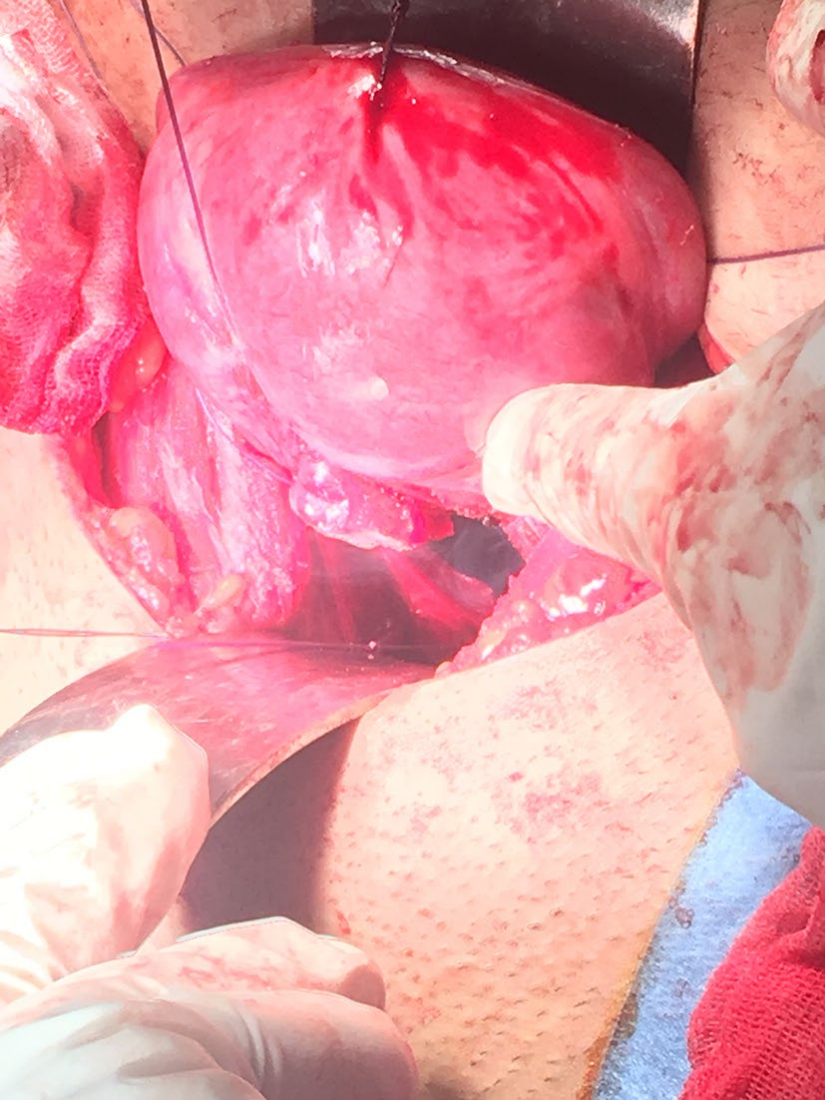
The cord-like cervix is excised

**Fig. 3:**
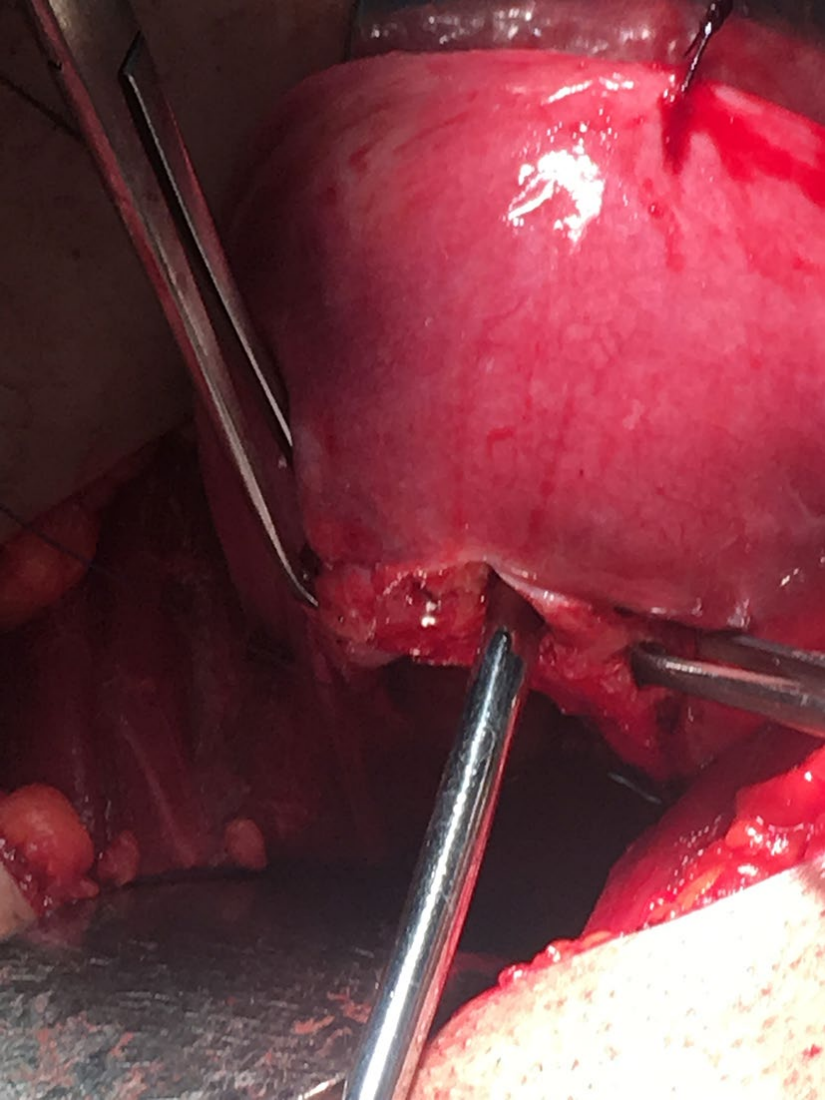
The uterine body is incised, and Hegar dilator is passed in the uterine cavity

**Fig. 4:**
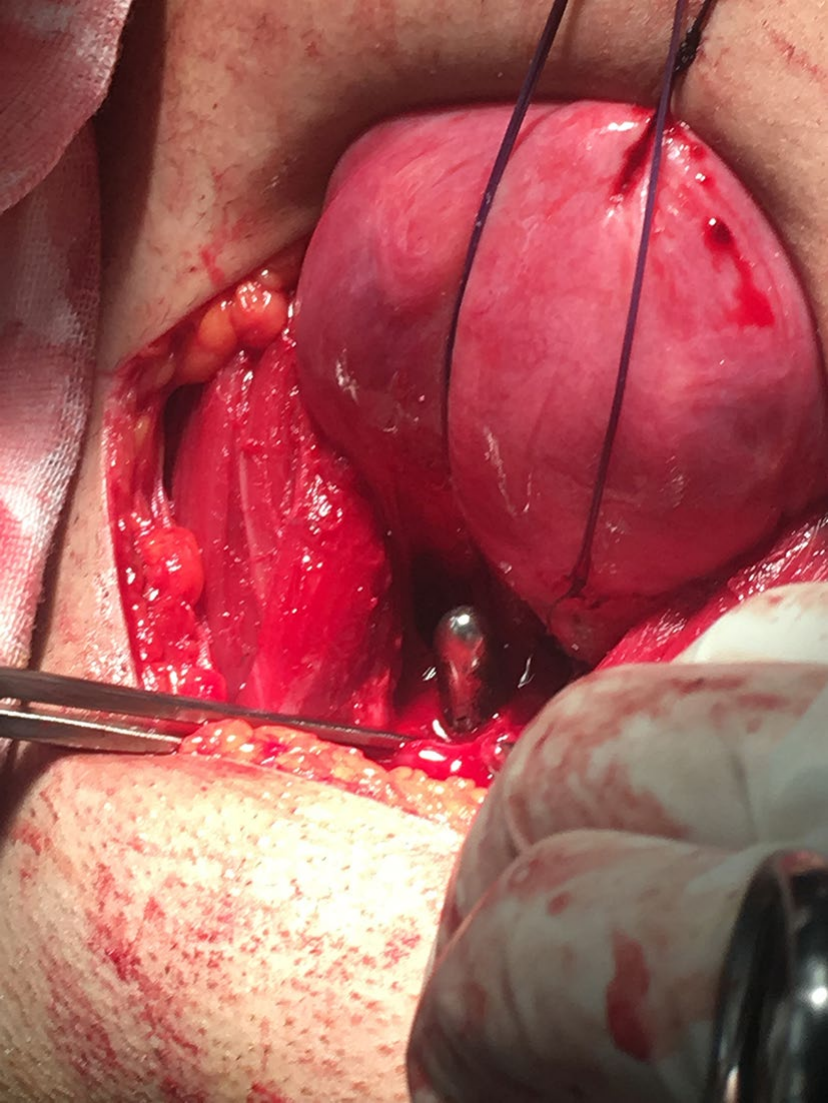
The dome of the vagina is opened, and Hegar dilator is passed through it from below upwards

**Fig. 5:**
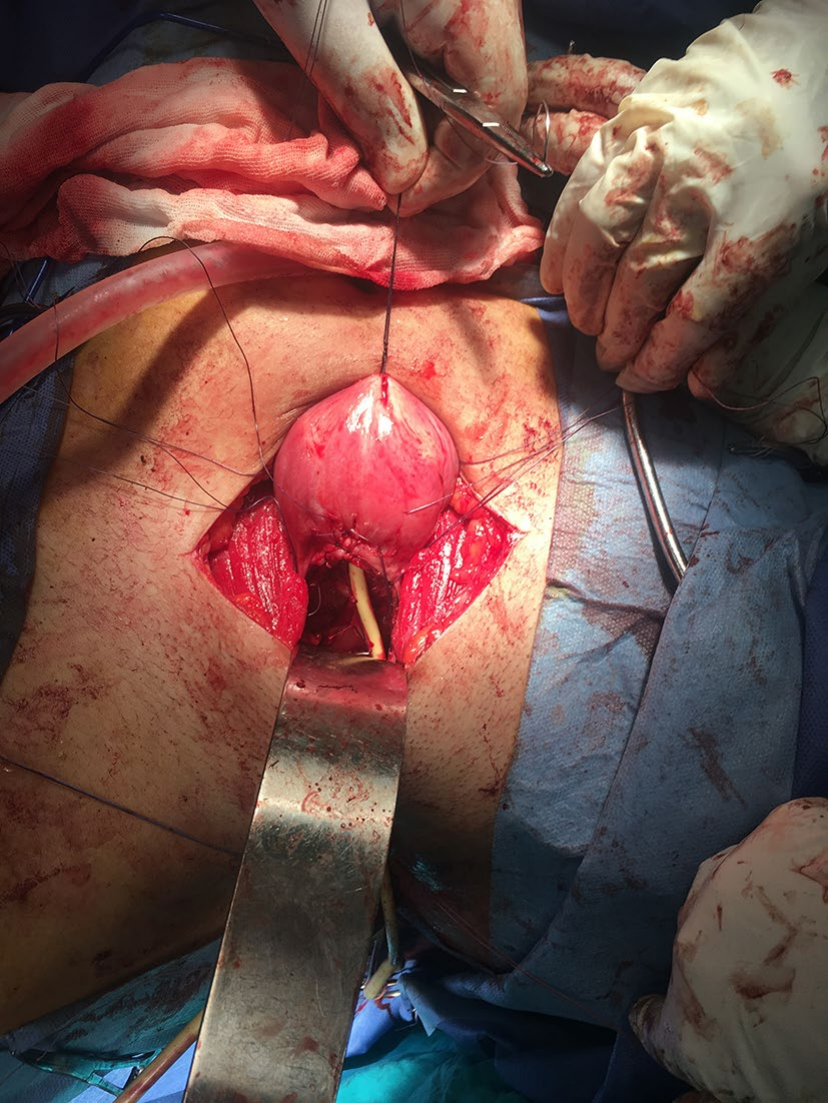
Foley’s catheter is passed from the vagina into the uterine cavity and pre-placed sutures are taken from the uterine incision to the vaginal incision

This is a retrospective cohort study. This study was performed in line with the principles of the Declaration of Helsinki. Nine patients who had cervical malformations with or without vaginal aplasia are included. Definitive diagnosis of the Müllerian anomalies was done by MRI. All patients underwent conservative surgeries tailored according to the existing Müllerian anomalies. Five patients had cervical agenesis with complete vaginal aplasia and underwent McIndoe’s vaginoplasty with split thickness skin graft and utero-vaginal anastomosis (UVA). Two patients had cervical agenesis with upper vaginal aplasia (vaginal length 4:5 cm) and underwent direct UVA without need for vaginoplasty. Two patients had cervical dysgenesis (obstruction) with normal vaginal length and underwent excision of the atretic part of the cervix then the anastomosis was performed (Figs. 1, 2, 3, 4, 5).

Utero-vaginal anastomosis was done by making 2 cm incision in the lower part of the anterior uterine wall then a transverse incision is made on the dome of the vagina/neovagina which is pushed upwards by 14 mm Hegar’s dilator. A urinary catheter is passed upwards through the vagina and guided in the uterine cavity and fixed by 3 ml saline in its balloon. Then, the edges of the uterine incision are sutured to the edges of the vaginal incision. Patients who had vaginal length < 4 cm were candidates for vaginoplasty by McIndoe’s technique using skin graft taken from the thigh. Cases who had cord-like cervical remnant, it is identified and excised first to allow approximation of the uterine body to the vaginal dome (Fig. 1:5).

Operations were performed at the department of Obstetrics and Gynecology of Cairo university teaching hospital between May 2015 and July 2019. Follow-up by transabdominal and/or transvaginal ultrasound was done monthly for 3 months postoperative then every 6 months thereafter. Data were analyzed after obtaining the approval from the institutional review board (IRB) of the faculty of medicine, Cairo University (N-60- 2022). The informed consent was waived by the IRB due to the retrospective nature of the study.

The following data were analysed:

- Occurrence of regular menstrual flow,
- Dysmenorrhea and its severity (mild /moderate/severe),

Mild (no need for analgesics)

Moderate (needs oral analgesics)

Severe (needs parenteral analgesics)

- Recurrence of hematometra,
- The need for dilatation of the anastomosis site and its time from the initial surgery,
- Number of the needed dilatations,
- The need for reoperation by repeating the steps of UVA,
- Infertility and its duration,
- Occurrence of pelvic inflammatory disease (PID),
- The need for assisted reproductive technology ( type, number of trials, difficulties, results)

### Statistical analysis

Data are presented as means (standard deviations), medians (quartiles), and frequencies (percentages) as appropriate.

## Results

Nine patients with cervical malformation are included in the study. All patients underwent conservative surgical management. Demographic and relevant preoperative and operative data are shown in Table 1.

**Table 1:** Demographic and preoperative data

	Age (at the primary surgery)	Utero-vaginal anomalies	Vaginal length before surgery (cm)	Operation
1	18	Cervical agenesis Vaginal aplasia	2	UVA^a^ and McIndoe’s vaginoplasty
2	21	Cervical agenesis Vaginal aplasia	2	UVA and McIndoe’s vaginoplasty
3	31	Cervical agenesis Vaginal aplasia	2	UVA and McIndoe’s vaginoplasty
4	17	Cervical agenesis Vaginal aplasia	2	UVA and McIndoe’s vaginoplasty
5	17	Cervical agenesis Vaginal aplasia	2	UVA and McIndoe’s vaginoplasty
6	22	Cervical agenesis Upper vaginal aplasia	4	UVA
7	20	Cervical agenesis Upper vaginal aplasia	5	UVA
8	27	Cervical obstruction	7	UVA
9	24	Cervical obstruction	8	UVA

^a^UVA utero-vaginal anastomosis

The median (quartiles) duration of follow-up is 36 (28.5–66) months. All patients achieved regular menstrual flow for variable durations (Table 2). As regards dysmenorrhea, four patients (44.4%) reported mild dysmenorrhea, four patients (44.4%) reported moderate dysmenorrhea while only one patient (12%) reported severe dysmenorrhea (Table 3).

**Table 2:** Follow-up data

	Follow-up duration (months)	Number of dilatations	Time of first dilatation from the primary surgery (months)	Number of needed reoperations	Time of reoperation from the primary surgery (months)	Duration of sexual activity (months)
1	36	1	14	1	15	18
2	27	0	No dilatation	0	–	17
3	34	4	12	0	–	32
4	30	4	10	1	13	0
5	60	0	No dilatation	0	–	28
6	45	1	36	1	44	4
7	82	1	24	0	–	0
8	15	1	10	0	–	12
9	72	5	12	0	–	70

**Table 3:** Demographic data and patient outcomes

Criterion	Values
Age (years) (*n* = 9)	21(17.5–25.5)
Follow-up duration (months) (*n* = 9)	36 (28.5–66)
Dysmenorrhea (*n* = 9) (frequency %) Mild Moderate Severe	4 (44%) 4 (44%) 1 (12%)
Time to first dilatation (months) (*n* = 7)	12 (10–24)
Patients who needed dilatation	7 (78%)
Number of dilatations	1 (1–4)
Patients who needed reoperation	3 (33%)
Time to reoperation (months) (*n* = 3)	24 ± 17
Pelvic inflammatory disease	2 (22%)
Sexual activity duration (months) (*n* = 7)	18(12–32)

Data are presented as median (quartiles), frequency (%), and mean ± standard deviation

Seven patients (78%) suffered from progressive stenosis of the anastomosis site which resulted in recurrence of hematometra and needed dilatation under anesthesia with ultrasound guidance. Three out of those seven patients needed repeated dilatation (4: 5 times) during their follow-up duration while the other four patients needed only one dilatation. We reported that the need for first dilatation is 12(10–24) months from the primary surgery. Three patients (33%) needed reoperation at a duration that ranged from 13 to 44 months from the primary surgery. Two patients (22%) did not need any surgical interventions and had regular menstruation for 27 months and 60-month follow-up durations. Two patients (22%) were complicated by pelvic inflammatory disease which responded to antibiotics regimens (Tables 2, 3).

Seven patients started regular sexual activity. The median (quartiles) duration of sexual activity is 18(12–32) months. All of them suffered from primary infertility.

Five of them sought intervention by ART. Two patients were not allowed to start the steps of in vitro fertilization (IVF) due to failure of mock passage of the embryo transfer catheter through the site of anastomosis. One of the patients who had UVA and McIndoe’s vaginoplasty underwent three trials of intracytoplasmic sperm injection (ICSI) ended with failure of conception. One patient who had cervical obstruction underwent two ICSI trials. The first trial was done 1 year after the primary operation and resulted in clinical pregnancy. Unfortunately, the pregnancy ended by missed abortion that needed hysteroscopic removal of the products of conception. The second trial was transfer of frozen embryos and ended by failure of conception. The other patient who had cervical obstruction underwent seven trials of ICSI which ended also by failure of conception. Surprisingly, this patient got pregnant spontaneously without any intervention after 6 years from her primary surgery and ended by live birth at 37 weeks gestation (Table 3).

Therefore, finally, clinical pregnancy was achieved in two patients (28.5%) out of seven and resulted in only one live birth (14%).

## Discussion

Transverse cervical malformations have been classified into cervical agenesis and cervical dysgenesis [5]. Cervical agenesis is complete absence of the cervix. Cervical dysgenesis is further categorized into cervical fragmentation, cervical fibrous cord and cervical obstruction [5]. Cervical malformations are also described as a spectrum. At one end of this spectrum are patients with a normal uterine body, hypoplastic cervix without a canal and a normal vagina. At the other end of the spectrum are patients with a uterine body, cervical agenesis but without vagina [6]. Conservative surgical management of such challenging conditions has three main goals: relief of the obstructive symptoms, achievement of satisfactory sexual function and preservation of the uterus for future fertility [1]. Assessment of these three goals requires long-term follow-up data which is lacking in the literature.

In this study, we present long-term results of conservative surgical management of nine cases of cervical malformations. Seven patients (78%) had cervical agenesis associated with either complete or upper vaginal aplasia while two patients had cervical dysgenesis in form of cervical obstruction with normal vaginal length. This agrees with what was mentioned in the literature that cervical agenesis is associated with upper or complete vaginal aplasia in 72% of cases, while in cases of cervical dysgenesis, vaginal aplasia is an unusual finding (3%)[7].

The age of the patients at the time of the primary UVA varied from 17 years up to 31 years. This is justified by the delayed diagnosis, mismanagement or receiving suppressive treatments such as gestagens.

Although all patients had relief of the obstructive symptoms and experienced regular menstrual flow, most of them (78%) experienced progressive stenosis at the site of anastomosis as a result of the natural healing process. This stenosis resulted in recurrence of hematometra which necessitated surgical intervention mainly by dilatation under anesthesia or reoperation. We reported that the need for first dilatation occurs mainly after 12 months postoperatively in cases who had McIndoe’s vaginoplasty and UVA, whereas the first dilatation was needed after longer duration (24 and 36 months) in cases who had UVA directly without vaginoplasty. From this observation, we recommend performing regular dilatation of the anastomosis site starting from 12 months after the operation and at regular intervals individualized according to the clinical evidence of stenosis. Dilatation should be done by Hegar dilators under ultrasound guidance after performing diagnostic hysteroscopy which paves the way by hydro-dissection and decreases the risk of false tracking. This agrees with Mikos et al. who recommended performing regular follow-up observation with hysteroscopy to confirm the accessibility of the cervical/neocervical conduit [1].

Regarding fertility, the results are somehow frustrating as all the seven sexually active patients suffered from primary infertility. Five of them sought help by ART. Clinical pregnancy was achieved only in two patients (28.5%) out of seven. Both patients had cervical obstruction with normal vagina while in the three patients of cervical agenesis the results were inferior. Two were not allowed to start IVF protocol due extremely stenosed anastomosis site that hindered mock examination by embryo transfer catheter while the third patient had repeated 2 ICSI failures.

In all ICSI trials done for the three patients, hysteroscopic examination and dilatation were performed immediately before starting the medication of the chosen protocol. No difficulty in ovum pick up was reported in all trials but embryo transfer was challenging due to the stenosed curved track in the patient who had UVA with McIndoe’s vaginoplasty. However, despite the adequate response to induction protocols and good quality of the transferred embryos, implantation failed repeatedly. The justification for this failure could be the impaired endometrial receptivity due to recurrent hematometra and increased intrauterine pressure during the obstruction periods. This agrees with Mikos et al. who suggested that uterine function is not always normal in these patients because in many cases, the diagnosis is delayed, and the uterine function is impaired because of the hematometra and the tubal function is affected by the severe pelvic adhesions already formed [1].

We did not offer abdominal cerclage for the patient who got pregnant. From our follow-up, we realized that the communication between the uterus and vagina/neovagina undergoes progressive stenosis that ends with tiny track for menstruation and the uterine body is just like a globular organ that is almost closed with no weak point to be reinforced by cerclage.

This is proven by the patient who completed 37 weeks gestation without rupture of membranes.

In a review article published in 2021, it is reported that 249 cases of cervical malformations were managed by different techniques of conservative surgeries and resulted in successful outcome by achieving menstruation in 228/249 patients (91.6%). Regarding fertility, 30 pregnancies (12.0%) were reported: 17 of 155 patients with cervical agenesis (11.0%) and 13 of 71 patients with cervical obstruction (18.3%) [8]. Unfortunately, in this review article, there was no information whether pregnancies occurred naturally or not and no information about the difficulties of the ART if used.

The limitation of our study is the small number of patients included because of the rarity of the condition.

From our study, we conclude that conservative surgical management of cervical malformations is a promising choice that can be offered with a main aim of relieving the obstructive symptoms and preserving the uterus for future fertility. Yet, the patients should be ready for a long journey of follow-up and regular dilatation of the anastomosis site starting from one year after the primary surgery. Primary infertility is expected while pregnancy is a remote hope that is hindered by many challenges. The results are worse in patients of cervical malformation associated with vaginal aplasia than in patient who had isolated cervical obstruction with normal vaginal length.

## Data Availability

Data of the study are available and can be provided upon request.
